# Diagnostic efficacy of ([Ca]×[Cl])/P combined with ALP in the diagnosis and subtype differentiation of primary hyperparathyroidism

**DOI:** 10.3389/fendo.2026.1790881

**Published:** 2026-06-05

**Authors:** Jingxian Zhao, Wei Chen, Jianping Lao, Donglian Wang, Tong Sun

**Affiliations:** Department of Clinical Laboratory, Taizhou Hospital of Zhejiang Province affiliated to Wenzhou Medical University, Taizhou, Zhejiang, China

**Keywords:** ([Ca]×[Cl])/P, ALP, diagnostic efficacy, PHPT, subtype differentiation

## Abstract

**Objective:**

To evaluate the diagnostic performance of the calcium-chloride/phosphorus ratio (([Ca]×[Cl])/P) combined with alkaline phosphatase (ALP) in primary hyperparathyroidism (PHPT) and its subtypes—hypercalcemic PHPT (HPHPT) and normocalcemic PHPT (NPHPT)—for accurate clinical differentiation.

**Methods:**

Clinical data of 135 surgically confirmed patients with PHPT (77 HPHPT, 58 NPHPT) and 135 age- and sex-matched healthy controls from Taizhou Enze Medical Center (Jan 2017–Dec 2024) were retrospectively analyzed. Serum ALP, Ca/P, Cl/P, and ([Ca]×[Cl])/P were measured; ROC curves assessed the diagnostic performance of single and combined indicators.

**Results:**

Most patients with PHPT were female. Adenoma prevalence was higher in the HPHPT group (83.12%) than in the NPHPT group (60.34%). ALP, parathyroid hormone (PTH), Ca/P, Cl/P, and ([Ca]×[Cl])/P levels were elevated in the HPHPT group compared with both the NPHPT and control groups, while serum phosphorus was lower (P<0.001). The area under the ROC curve (AUC) for ([Ca]×[Cl])/P in diagnosing PHPT was 0.960. Combined detection of ([Ca]×[Cl])/P (250.67 mmol/L) and ALP (93.50 U/L) yielded an AUC of 0.983 (sensitivity: 94.8%, specificity: 98.5%, accuracy: 95.0%), comparable to that of PTH (AUC = 0.997). The AUC for differentiating HPHPT from NPHPT was 0.881, and for distinguishing adenomas from hyperplasia was 0.677, demonstrating superior subtype discrimination compared with other indicators, including PTH.

**Conclusion:**

The combination of ([Ca]×[Cl])/P and ALP demonstrates favorable sensitivity and specificity for the diagnosis of PHPT and the differentiation of HPHPT from NPHPT, and exhibits clinical practicality. In addition, it provides supplementary value in distinguishing parathyroid adenoma from hyperplasia.

## 1 Introduction

Primary hyperparathyroidism (PHPT) is characterized by excessive secretion of parathyroid hormone (PTH) due to lesions in one or more of the four parathyroid glands (1). Clinical manifestations typically include bone pain, fractures, and urolithiasis, along with non-specific symptoms, such as fatigue and hypertension. PHPT can be categorized into two subtypes: hypercalcemic PHPT (HPHPT) and normocalcemic PHPT (NPHPT). Patients with NPHPT are often asymptomatic, exhibiting only mild hypercalcemia, which may lead to underdiagnosis (2). Pathologically, PHPT is primarily classified as parathyroid adenoma (PA) or parathyroid hyperplasia (PH). Despite advances in imaging technologies, standardized morphological criteria to reliably differentiate adenomas from hyperplasia remain lacking (3). Currently, in resource-limited primary care hospitals with technological and equipment constraints, PTH measurement may not be widely available; thus, diagnosis predominantly depends on electrolyte assessments and routine biochemistry.

Recent studies have suggested that the calcium-chloride/phosphorus ratio (([Ca]×[Cl])/P) serves as a useful diagnostic marker for NPHPT (4), while alkaline phosphatase (ALP) can reflect bone metabolism in patients with HPHPT. However, the diagnostic utility of combining ([Ca]×[Cl])/P and ALP for PHPT diagnosis and subtype differentiation has not been adequately explored. Therefore, this retrospective study aimed to analyze clinical data of patients with PHPT to evaluate the combined diagnostic performance of ([Ca]×[Cl])/P and ALP, thereby providing an effective scheme for early screening and classification of PHPT.

## 2 Materials and methods

### 2.1 Study participants

**Figure 1:**
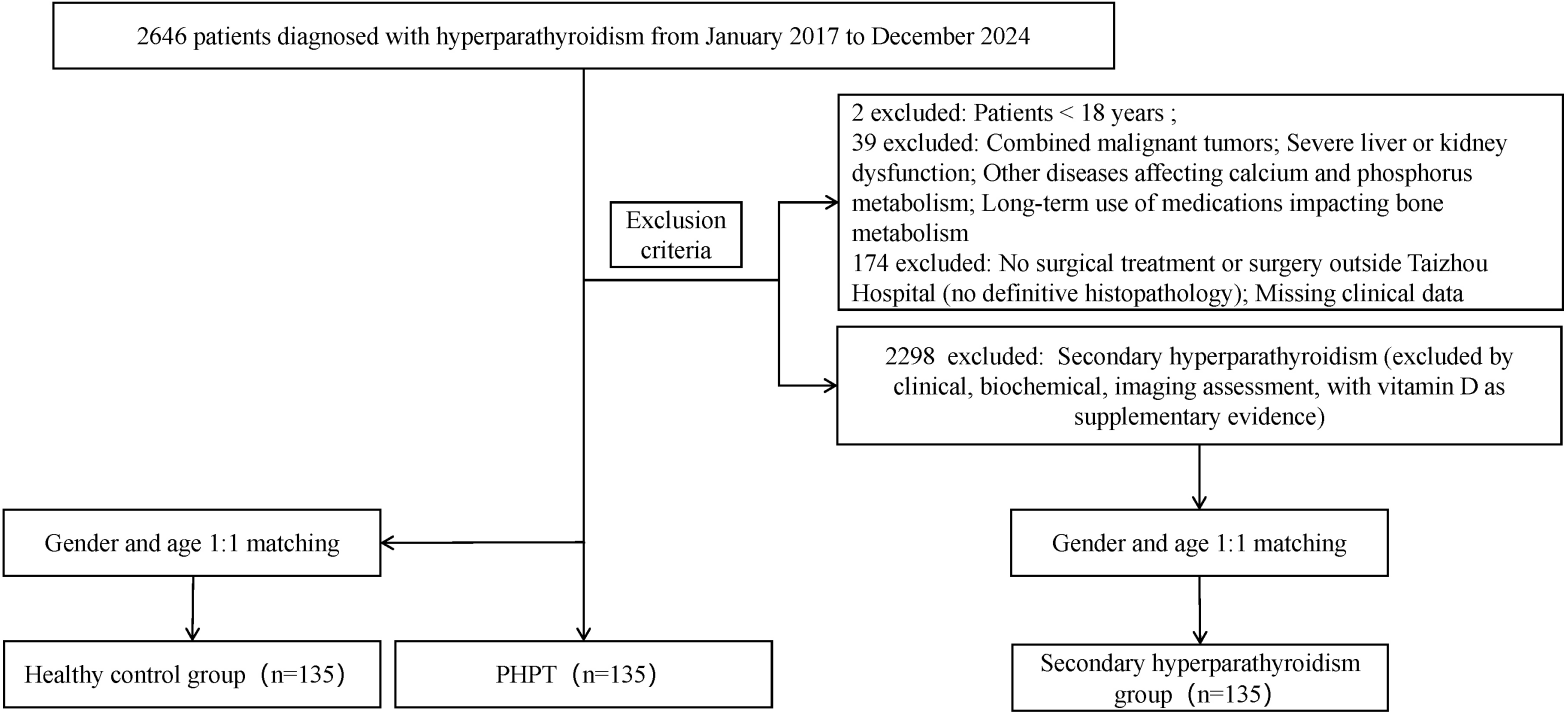
The process of the selection criteria.

This retrospective study included 135 patients diagnosed with PHPT who underwent surgical resection with pathological confirmation at three campuses of Taizhou Hospital between January 2017 and December 2024. In accordance with the 2022 World Health Organization (WHO) classification of parathyroid tumors (5), no atypical parathyroid tumor or parathyroid carcinoma was documented in the 135 patients upon pathological examination. Surgical indications followed the Fifth International Workshop guidelines (6); symptomatic patients met criteria for surgery, and asymptomatic patients underwent surgery either on physician recommendation or at their own request for curative resection. According to guideline criteria (3, 7), Patients were divided into two groups based on albumin-corrected serum calcium levels: the HPHPT group, with albumin-corrected calcium > 2.60 mmol/L (n =77), and the NPHPT group, with albumin-corrected calcium ranging from 2.12–2.60 mmol/L (n = 58). A total of 135 healthy controls matched by sex and age were included in this study, and 135 patients with secondary hyperparathyroidism were additionally enrolled as a differential diagnosis control group. The sample size was determined based on the actual availability of eligible cases consecutively recruited at our hospital during the study period to ensure sufficient statistical power for between-group comparisons. Controls were matched with patients at a 1:1 ratio according to sex and age to reduce potential confounding factors. All subjects in the control group were confirmed by detailed screening to have normal renal function indicators, no history of long-term use of drugs affecting calcium-phosphorus metabolism, and no history of skeletal system diseases, endocrine diseases or malignant tumors. The exclusion criteria for patients with PHPT comprised age < 18 years; failure to receive surgical treatment or undergo surgery at Taizhou Hospital, for whom definitive histopathological confirmation could not be obtained; secondary hyperparathyroidism was excluded by clinicians according to relevant guidelines, using comprehensive clinical, biochemical and imaging assessments, and vitamin D data were employed as supplementary evidence where available (8); presence of combined malignant tumors; severe liver or kidney dysfunction; other diseases affecting calcium and phosphorus metabolism; long-term use of medications impacting bone metabolism; and missing clinical data (**Figure 1**).

### 2.2 Data collection

Routine blood and biochemical test results were collected from all patients. Preoperative clinical indicators analyzed included liver function tests —alanine aminotransferase (ALT), aspartate aminotransferase (AST), alkaline phosphatase (ALP), total bilirubin (TB), direct bilirubin (DB), total protein (TP), and albumin (ALB)—as well as electrolytes including potassium (K), sodium (Na), chloride (Cl), calcium (Ca), and phosphorus (P), in addition to routine blood counts and parathyroid hormone (PTH). PTH and serum Ca levels were also measured 0–1 day postoperatively. Liver function, renal function, and electrolyte levels were measured using a Beckman automatic biochemical analyzer (AU5821, Beckman Coulter, Brea, CA, USA). 25-hydroxyvitamin D [25(OH)D] was measured using an AB Sciex liquid chromatography-tandem mass spectrometry (LC-MS/MS) system. PTH was measured via a Roche automatic electrochemiluminescence immunoanalyzer (e801, cobas, USA), and routine blood tests were performed using a Sysmex automatic hematology analyzer (XN2800, Sysmex, Japan). Bone mineral density (BMD) was measured using a GE Healthcare dual-energy X-ray absorptiometry (DXA) instrument. Throughout the study period, all detections were consistently performed using the same aforementioned instruments and calibration standards, and all instruments were maintained and calibrated regularly. Quality control procedures were performed daily for each test, and all assays consistently achieved excellent results in the inter-laboratory quality assessment program organized by the National Health Commission of the People’s Republic of China. Plasma albumin-adjusted corrected Ca levels were calculated as follows: Albumin-corrected calcium (mmol/L) = total serum calcium (mmol/L) + 0.02×(40-patient’s serum albumin concentration (g/L)) (9); ([Ca]×[Cl])/P (mmol/L) =(albumin-corrected calcium × serum chloride)/serum phosphorus.

The reference ranges used for adults were as follows: ALT: male: 9–50 (U/L), female: 7–40 (U/L); AST: male: 15–40 (U/L), female: 13–35 (U/L); ALP: male: 45–125 (U/L), female: 35–135 (U/L); TB: 3.4–20.5 (μmol/L); DB: ≤4.0 (μmol/L); TP: 65–85 (g/L); ALB: 35.0–55.0 (g/L); eGFR:≥90 (mL/min/1.73m^2^); K:3.5–5.3 (mmol/L); NA:137.0–147.0 (mmol/L); CL:99–110 (mmol/L); CA: 2.12–2.60 (mmol/L); P: 0.85–1.51(mmol/L); 25(OH)D: deficient <12.00 ng/mL, insufficient ≥12.00 to <20.00 ng/mL, sufficient ≥20.00 ng/mL; 24-hour urinary calcium: 2.50–7.50 mmol/24h; PTH: 15–65 (pg/ml); WBC: 3.95–9.50 (×10^9^/L); RBC: male: 4.30–5.80 (×10^12^/L), female:3.80–5.10 (×10^12^/L); and PLT:125-35 (×10^9^/L). BMD T-score: normal ≥−1.0, osteopenia −2.5 to <−1.0, osteoporosis ≤−2.5.

### 2.3 Statistical analysis

Measurement data were expressed as mean ± standard deviation or median (M)with interquartile range (IQR). The Shapiro–Wilk test was used to assess the normality of data distribution. For inter-group difference analysis, an independent samples t-test was used for normally distributed variables, and the Mann–Whitney U test for non-normally distributed data. Multiple group comparisons were conducted using the Kruskal–Wallis H test. Diagnostic performance of each indicator was evaluated using the Receiver Operating Characteristic (ROC) curve analysis. With 95% confidence intervals (CI) for all area under the curve (AUC) values reported. Additionally, statistical analyses were performed using IBM SPSS version 22.0 software, while graphical presentations were prepared with Origin 2019b and R. A p-value < 0.05 was considered statistically significant.

### 2.4 Sample size determination and *post-hoc* power analysis

A total of 135 patients with PHPT and 135 controls were enrolled in this 1:1 case-control study. *Post-hoc* power analysis was performed using G*Power 3.1.9.7 software to verify the statistical adequacy of the sample size. For the comparison of continuous variables between the two groups, a two-independent-samples t-test was applied. Assuming a medium effect size (d = 0.5), a one-sided type I error rate of α= 0.05, and 135 subjects in each group, the achieved statistical power was 0.9929, indicating that the sample size was sufficient to minimize the risk of type II error. For the diagnostic performance analysis of the combined indicator, a logistic regression model was used. Assuming a medium effect size (odds ratio = 2.0), a two-sided type I error rate of α = 0.05, a baseline event rate of 0.5, and a total sample size of 270, the achieved statistical power was 0.9997, indicating that the sample size was sufficient to ensure the reliability of the diagnostic analysis.

## 3 Results

### 3.1 Demographic and clinical characteristics of patients with PHPT

Among the 270 patients, no significant difference was observed in sex distribution among the HPHPT (n=77), NPHPT (n=58), and healthy control (n=135) groups (P = 0.529). However, females predominated across all groups, accounting for 68.83%, 77.59%, and 72.59% of each group, respectively. Additionally, median age and body mass index (BMI) did not differ significantly among the groups. The prevalence of hypertension was significantly higher in both the HPHPT and NPHPT groups than in the healthy control group (P<0.05), although no significant difference was observed between the two PHPT groups. The prevalence of diabetes mellitus and kidney stones did not differ significantly among the three groups.

The proportion of adenomas in the HPHPT group (83.12%) was significantly higher than in the NPHPT group (60.34%, P<0.003). Overall, adenomas accounted for 57.04% (77/135) of lesions, while hyperplasia accounted for 42.96% (58/135). The proportion of single-gland lesions was 81.82% in the HPHPT group and 86.21% in the NPHPT group, resulting in a combined rate of 83.70% (113/135).

Levels of ALP, corrected calcium, and PTH were significantly elevated in the HPHPT group compared to the NPHPT and control groups, with significant differences also observed between the NPHPT and control groups (P<0.001). For the calcium-phosphorus ratio (Ca/P), chloride-phosphorus ratio (Cl/P), and ([Ca]×[Cl])/P composite index, levels were the highest in the HPHPT group, followed by the NPHPT group, and the lowest in the control group, with statistically significant differences between all pairs of groups (P<0.001). Postoperative decreases in PTH and calcium levels were significantly greater in the HPHPT group, with median reductions of 95.10% and 17.96%, respectively, compared to 81.13% and 12.86% in the NPHPT group (**Table 1**).

**Table 1:** Baseline characteristics and laboratory indicators of patients with primary hyperparathyroidism and healthy controls.

Variable	HPHPT (n=77)	NPHPT (n=58)	Control (n=135)	P value
Gender (Male/Female)				0.529
Male n (%)	24 (31.17)	13 (22.41)	37 (27.41)	
Female n (%)	53 (68.83)	45 (77.59)	98 (72.59)	
Median age (mean,sd), year	53.45 ± 12.17	53.43 ± 11.34	53.15 ± 13.09	0.964
BMI (IQRa), kg/m2	23.09 ± 6.06	24.58 ± 3.96	23.23 ± 2.48	0.170
Hypertension n (%)	26 (33.76)	18 (31.03)	16 (11.85)^#+^	<0.001
Diabetes n (%)	9 (11.69)	7 (12.07)	8 (5.93)	0.231
Kidney stones n (%)	15 (19.48)	5 (8.62)	27 (20.0)	0.137
Maximum diameter (mm)	21.68 ± 12.72	21.44 ± 7.46	/	0.282
Lesions				0.494
Single-gland lesion	63 (81.82)	50 (86.21)	/	
Multiglandular lesions	14 (18.18)	8 (13.79)	/	
Pathological results				0.003
Adenoma n (%)	64 (83.12)	35 (60.34)	/	
Hyperplasia n (%)	13 (16.88)	23 (39.66)	/	
Laboratory indicators				
PTH (pg/mL)	263.3 (189.0-531.0)	129.3 (92.8-186.6)*	34.2 (28.4-44.0)^#+^	<0.001
WBC (×10^9/L)	6.1 (4.8-7.7)	6.0 (4.8-7.0)	5.7 (4.8-6.5)^#^	0.091
RBC (×10^12/L)	4.54 (4.15-4.89)	4.57 (4.16-4.90)	4.58 (4.31-4.92)	0.349
PLT (×10^9/L)	231.0 (195.0-298.0)	238.5 (208.8-298.8)	235.0 (202.0-274.0)	0.433
ALT (U/L)	18.0 (14.1-30.0)	19.5 (13.8-34.5)	16.0 (13.0-23.0)	0.119
AST (U/L)	22.0 (17.0-26.5)	23.0 (19.0-28.0)*	21.0 (18.0-26.0)^+^	0.091
ALP (U/L)	141.0 (107.0-234.5)	102.0 (81.8-119.0)*	68.0 (56.0-80.0)^#+^	<0.001
TP (g/L)	72.2 (68.7-75.2)	75.2 (70.4-77.7)	73.0 (70.6-75.4)	0.051
ALB (g/L)	44.1 (41.8-45.7)	45.8 (43.6-48.0)*	46.5 (44.8-48.2)^#^	<0.001
eGFR (mL/min/1.73m²)	98.0 (77.0-110.5)	100.0 (91.0-110.8)	102.0 (92.0-110.0)	0.054
K (mmol/L)	4.16 (3.94-4.44)	4.15 (4.00-4.38)	4.07 (3.87-4.21)^#+^	0.006
NA (mmol/L)	140.2 (138.7-141.6)	140.2 (138.4-141.7)	140.0 (138.6-141.3)	0.746
CL (mmol/L)	107.6 (105.6-109.5)	105.7 (104.8-107.9)*	104.6 (103.3-105.8)^#^	<0.001
P (mmol/L)	0.73 (0.64-0.82)	0.90 (0.76-1.02)*	1.12 (1.00-1.21)^#+^	<0.001
Albumin-corrected calcium (mmol/L)	2.84 (2.75-3.02)	2.49 (2.37-2.59)*	2.17 (2.13-2.23)^#+^	<0.001
Ca/P	3.90 (3.42-4.74)	2.82 (2.38-3.29)*	1.98 (1.80-2.18)^#+^	<0.001
CL/P	145.38 (129.75-169.24)	120.63 (101.92-139.37)*	94.30 (86.61-104.60)^#+^	<0.001
([Ca]×[Cl])/P (mmol/L)	428.13 (367.13-509.84)	302.25 (250.54-350.70)*	207.37 (188.68-227.29)^#+^	<0.001
Postoperative 0-1 day PTH (pg/mL)	12.9 (7.8-25.1)	24.4 (13.2-36.2)	/	<0.001
Postoperative 0-1 day calcium (mmol/L)	2.33 (2.20-2.46)	2.17 (2.11-2.29)	/	<0.001

Note: Data are expressed as median (IQR), n (%), or mean ± sd. *: P < 0.05 (HPHPT vs. NPHPT); #: P < 0.05 (HPHPT vs. control group); +: P < 0.05 (NPHPT vs. control group); Bold values indicate statistically significant differences among the three groups (P < 0.05); P: P value for comparison among HPHPT, NPHPT, and control groups; BMI: Body Mass Index; PTH: Parathyroid Hormone; WBC: White Blood Cell; RBC: Red Blood Cell; PLT: Platelet; ALT: Alanine Aminotransferase; AST: Aspartate Aminotransferase; ALP: Alkaline Phosphatase; TP: Total Protein; ALB: Albumin; eGFR: Estimated glomerular filtration rate; Ca/P: albumin-Corrected Calcium to Phosphorus Ratio; Cl/P: chloride to Phosphorus Ratio; ([Ca]×[Cl])/P: the ratio of the product of albumin-corrected calcium and chloride to phosphorus.

Secondary hyperparathyroidism patients showed the highest levels of PTH, phosphorus, and alkaline phosphatase, whereas patients with HPHPT had the highest levels of corrected calcium, Ca/P, Cl/P, and ([Ca]×[Cl])/P, with all differences being statistically significant (P < 0.001) (**Supplementary Table 1**).

### 3.2 Distribution characteristics of calcium and phosphorus metabolism-related indicators in HPHPT, NPHPT, and control groups

**Figure 2:**
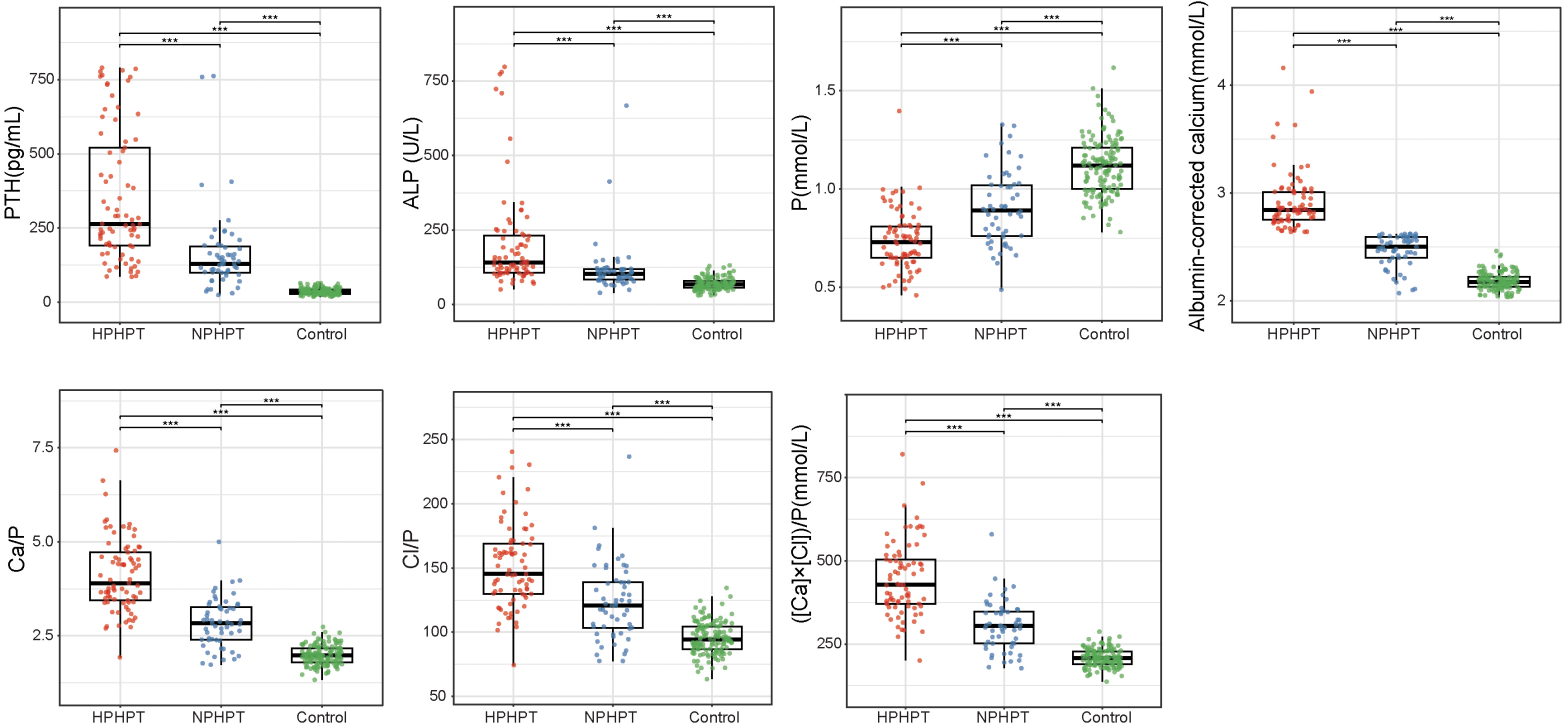
Distribution characteristics of calcium and phosphorus metabolism-related indicators in HPHPT, NPHPT, and control groups. Note: ***: *P*<0.001; PTH: Parathyroid hormone; ALP: Alkaline phosphatase; Albumin-corrected calcium (mmol/L) = total serum calcium (mmol/L) + 0.02×(40-patient’s serum albumin concentration (g/L)); Ca/P : albumin-corrected calcium to phosphorous ratio; Cl/P: chloride to phosphorus ratio; ([Ca]×[Cl])/P: the ratio of the product of albumin-corrected calcium and chloride to phosphorus.

Significant differences among the three groups were observed for PTH, ALP, albumin-corrected calcium, Ca/P, Cl/P, and ([Ca]×[Cl])/P (P < 0.001), with the highest values in the HPHPT group, followed by the NPHPT and the control groups. Conversely, serum phosphorus was lowest in the HPHPT group and highest in the control group, with significant differences across groups (P < 0.001) (**Figure 2**).

### 3.3 Comparisons of 25(OH)D, 24-hour urinary calcium, and BMD T-score across subgroups of PHPT and individual data of 15 complete cases

**Figure 3:**
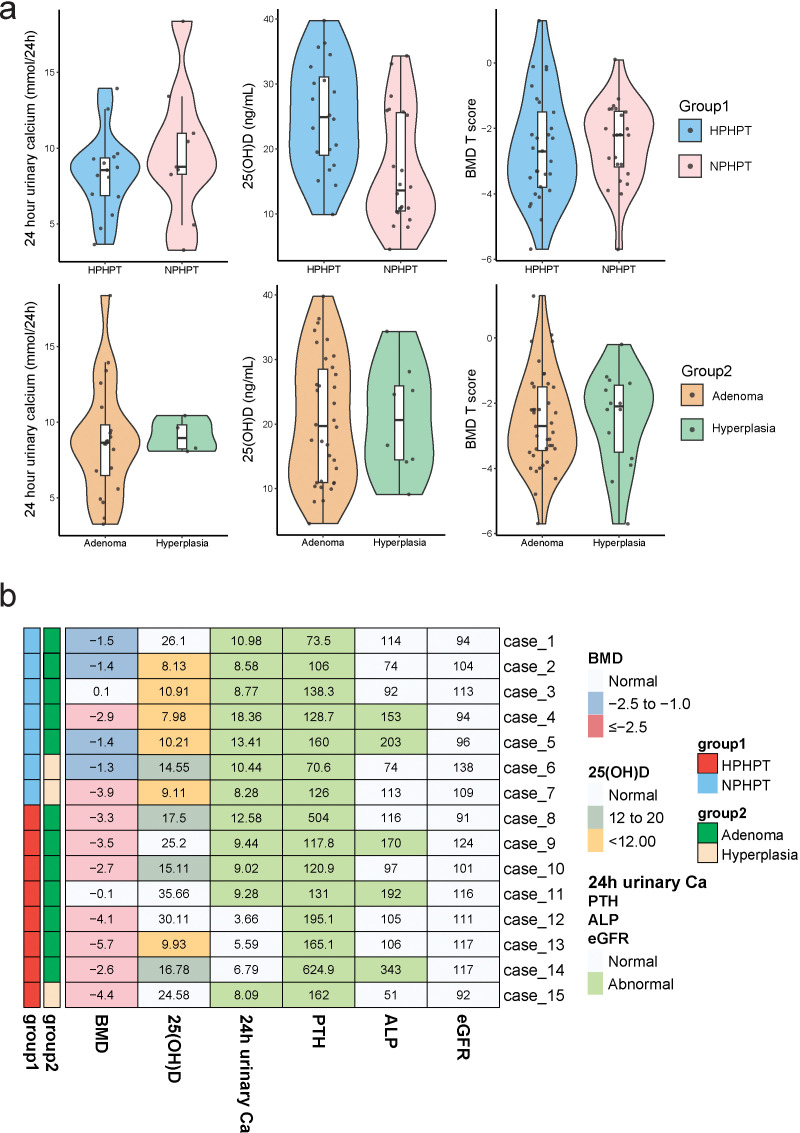
Comparisons of 25(OH)D, 24-hour urinary calcium, and BMD T-score across subgroups of PHPT and individual data of 15 complete cases. **(a)** Violin plots showing 25(OH)D, 24-hour urinary calcium, and BMD T-score between HPHPT and NPHPT, and between adenoma and hyperplasia. **(b)** Individual distributions of 25(OH)D, 24-hour urinary calcium, BMD T-score, PTH, ALP, and eGFR in 15 patients with complete data.

Patients with PHPT were subgrouped by clinical phenotype and pathological type. Owing to missing data, the current analysis included 42 valid cases for 25(OH)D, 24 valid cases for 24-hour urinary calcium, and 53 valid cases for BMD T-score. Individual data were presented for 15 cases with complete measurements. According to clinical classification, HPHPT exhibited higher 24-hour urinary calcium levels, lower 25(OH)D levels, and lower BMD T-scores compared with NPHPT. By pathological type, no significant differences were found in the distribution of 25(OH)D, 24-hour urinary calcium, or BMD T-scores between parathyroid adenoma and parathyroid hyperplasia (**Figure 3a**).

Among the 15 cases with complete data, most patients had 25(OH)D insufficiency or deficiency, 24-hour urinary calcium varied considerably, and BMD T-scores were mostly in the range of osteopenia or osteoporosis, accompanied by elevated PTH; some patients had increased ALP, while eGFR was generally normal (**Figure 3b**).

### 3.4 Diagnostic and differential performance of ([Ca]×[Cl])/P combined with ALP in HPHPT, NPHPT, control groups, and different subtypes

**Figure 4:**
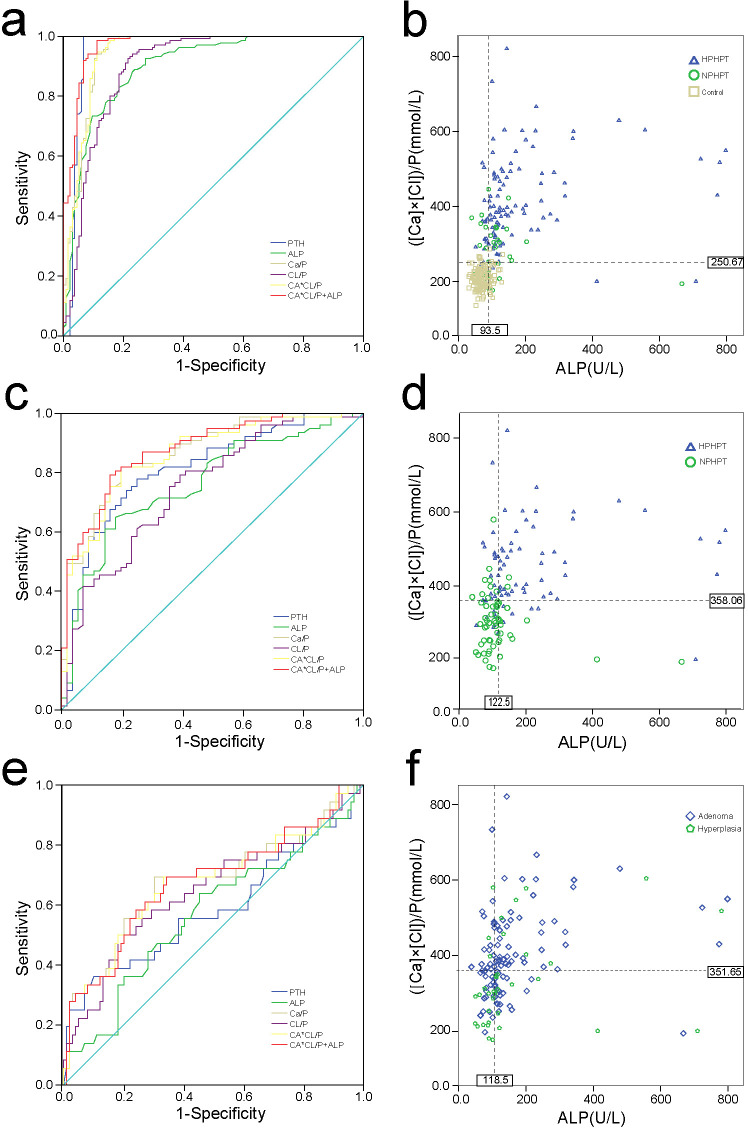
ROC curves and diagnostic efficacy analysis of PTH, ALP, Ca/P ratio, Cl/P ratio, ([Ca]×[Cl])/P ratio, and ([Ca]×[Cl])/P + ALP between PHPT and control groups, HPHPT and NPHPT groups, and adenoma and hyperplasia groups. **(a, b)** ROC analysis and biomarker distribution in PHPT and controls; **(c, d)** ROC analysis and biomarker distribution in HPHPT and NPHPT; **(e, f)** ROC analysis and biomarker distribution in adenoma and hyperplasia. PTH, Parathyroid hormone; ALP, Alkaline phosphatase; Ca/P, albumin-corrected calcium to phosphorous ratio; Cl/P, chloride to phosphorus ratio; ([Ca]×[Cl])/P, the ratio of the product of albumin-corrected calcium and chloride to phosphorus.

**Table 2** and **Figure 4** summarize the area under the ROC curve (AUC), sensitivity, specificity, positive predictive value (PPV), negative predictive value (NPV), and accuracy of PTH, ALP, Ca/P, Cl/P, ([Ca]×[Cl])/P, and combined ([Ca]×[Cl])/P and ALP detection for three key comparisons: PHPT vs. controls, HPHPT vs. NPHPT, and adenoma vs. hyperplasia.

**Table 2:** Diagnostic efficacy of each indicator and combined detection of ([Ca]×[Cl])/P + ALP in differentiating PHPT vs control, HPHPT vs NPHPT, and adenoma vs hyperplasia (AUC, sensitivity, specificity, PPV, NPV, and accuracy).

Comparison	Variable	Cut-off	AUC (95% CI)	Sensitivity	Specificity	PPV	NPV	Accuracy
PHPT vs control	PTH (pg/mL)	69.15	0.997 (0.994-1.000)	0.978	1.000	1.00	0.96	0.98
	ALP (U/L)	93.50	0.907 (0.871-0.943)	0.785	0.896	0.87	0.79	0.83
	Ca/P	2.57	0.960 (0.933-0.986)	0.896	0.978	0.97	0.87	0.91
	CL/P	114.36	0.910 (0.872-0.948)	0.807	0.940	0.93	0.80	0.86
	([Ca]×[Cl])/P	250.67	0.960 (0.934-0.987)	0.933	0.948	0.94	0.90	0.92
	([Ca]×[Cl])/P+ALP	–	0.983 (0.967-0.999)	0.948	0.985	0.98	0.93	0.95
HPHPT vs control	PTH (pg/mL)	–	1.000	1.000	1.000	1.00	1.00	1.00
	ALP	–	0.950 (0.919-0.981)	0.909	0.889	0.82	0.94	0.90
	Ca/P	–	0.992 (0.978-1.000)	0.987	0.993	0.96	0.99	0.98
	CL/P	–	0.967 (0.939-0.995)	0.909	0.941	0.90	0.95	0.93
	([Ca]×[Cl])/P	–	0.992 (0.977-1.000)	0.987	0.985	0.92	0.99	0.96
	([Ca]×[Cl])/P+ALP	–	0.999 (0.998-1.000)	0.992	0.985	0.97	1.00	0.99
NPHPT vs control	PTH (pg/mL)	–	0.951 (0.907-0.996)	0.891	1.000	1.00	0.96	0.97
	ALP	–	0.834 (0.769-0.899)	0.845	0.733	0.69	0.83	0.79
	Ca/P	–	0.887 (0.828-0.946)	0.776	0.926	0.93	0.87	0.88
	CL/P	–	0.810 (0.734-0.886)	0.621	0.926	0.81	0.84	0.83
	([Ca]×[Cl])/P	–	0.889 (0.830-0.949)	0.759	0.948	0.86	0.90	0.89
	([Ca]×[Cl])/P+ALP	–	0.934 (0.893-0.974)	0.845	0.919	0.93	0.93	0.93
Adenoma vs control	PTH (pg/mL)	–	0.998 (0.995-1.000)	0.980	1.000	1.00	0.99	1.00
	ALP	–	0.921 (0.886-0.956)	0.816	0.888	0.84	0.86	0.85
	Ca/P	–	0.972 (0.946-0.998)	0.951	0.940	0.92	0.97	0.95
	CL/P	–	0.928 (0.891-0.965)	0.845	0.933	0.91	0.89	0.90
	([Ca]×[Cl])/P	–	0.973 (0.947-0.998)	0.951	0.948	0.93	0.97	0.95
	([Ca]×[Cl])/P+ALP	–	0.983 (0.965-1.000)	0.942	0.985	0.98	0.98	0.98
Hyperplasia vs control	PTH (pg/mL)	–	0.926 (0.856-0.996)	0.853	1.000	1.00	0.96	0.97
	ALP	–	0.854 (0.773-0.936)	0.833	0.807	0.62	0.91	0.84
	Ca/P	–	0.855 (0.771-0.938)	0.667	0.993	0.89	0.92	0.91
	CL/P	–	0.800 (0.696-0.904)	0.696	0.904	0.72	0.89	0.87
	([Ca]×[Cl])/P	–	0.855 (0.771-0.940)	0.667	0.993	0.78	0.92	0.89
	([Ca]×[Cl])/P+ALP	–	0.915 (0.857-0.973)	0.778	0.941	0.90	0.95	0.94
HPHPT vs NPHPT	PTH (pg/mL)	185.80	0.808 (0.731-0.884)	0.779	0.745	0.81	0.72	0.77
	ALP	122.50	0.769 (0.689-0.850)	0.649	0.827	0.83	0.64	0.73
	Ca/P	3.36	0.818 (0.810-0.929)	0.818	0.810	0.85	0.77	0.81
	CL/P	126.51	0.758 (0.676-0.839)	0.792	0.621	0.73	0.69	0.72
	([Ca]×[Cl])/P	358.06	0.865 (0.804-0.926)	0.818	0.810	0.85	0.77	0.81
	([Ca]×[Cl])/P+ALP	–	0.881 (0.825-0.937)	0.792	0.845	0.96	0.83	0.89
Adenoma vs Hyperplasia	PTH (pg/mL)	102.25	0.587 (0.470-0.705)	0.908	0.324	0.80	0.59	0.76
	ALP	118.50	0.569 (0.453-0.685)	0.545	0.639	0.81	0.34	0.57
	Ca/P	3.24	0.676 (0.561-0.791)	0.697	0.694	0.86	0.45	0.69
	CL/P	117.35	0.647 (0.532-0.763)	0.818	0.500	0.82	0.50	0.73
	([Ca]×[Cl])/P	351.65	0.675 (0.561-0.790)	0.675	0.667	0.86	0.43	0.67
	([Ca]×[Cl])/P+ALP	–	0.677 (0.563-0.790)	0.657	0.694	0.84	0.50	0.69

PTH, Parathyroid hormone; ALP, Alkaline phosphatase; Ca/P, albumin-corrected calcium to phosphorous ratio; Cl/P, chlorideto phosphorusratio; ([Ca]×[Cl])/P, the ratio of the product of albumin-corrected calcium and chloride to phosphorus.

NPV, negative predictive value; PPV, positive predictive value.

In the overall cohort, ROC analysis for PHPT diagnosis showed that ([Ca]×[Cl])/P had an AUC of 0.960 (95%CI: 0.934–0.987), comparable to that of Ca/P (AUC 0.960; 95%CI: 0.933–0.986) and significantly superior to that of Cl/P (AUC 0.910; 95% CI: 0.872–0.948). ALP alone also demonstrated good diagnostic value (AUC 0.907; 95%CI: 0.871–0.943). Notably, combining ([Ca]×[Cl])/P and ALP further improved the diagnostic efficacy, increasing the AUC to 0.983 (95% CI: 0.967–0.999), approaching that of PTH (AUC 0.997; 95% CI: 0.994–1.000) (**Figure 4a**). Using cut-off values of 250.67 mmol/L for ([Ca]×[Cl])/P and 93.50 U/L for ALP, the sensitivity, specificity, PPV, NPV, and accuracy were 94.8%, 98.5%, 98.0%, 93.0%, and 95.0%, respectively (**Figure 4b**).

Further analysis of the diagnostic differences of combined detection in different subtypes and pathological types of PHPT showed that the AUC for differentiating HPHPT from the healthy control group was 0.999 (95%CI = 0.998–1.000), which was significantly superior to that for differentiating NPHPT from the healthy control group (0.934, 95%CI = 0.893–0.974). Meanwhile, the AUC for differentiating adenoma from the healthy control group was 0.983 (95%CI = 0.965–1.000), which was also superior to that for differentiating hyperplasia from the healthy control group (0.915, 95%CI = 0.857–0.973).

In differentiating between the HPHPT and NPHPT groups, combined detection of ([Ca]×[Cl])/P and ALP demonstrated the highest diagnostic efficacy, with an AUC of 0.881 (95% CI = 0.825–0.937), outperforming single indicators such as Ca/P, Cl/P, ALP, and even PTH (**Figure 4c**). Using cut-off values of 358.06 mmol/L for ([Ca]×[Cl])/P and 122.50 U/L for ALP, the combined detection yielded a PPV of 0.96, NPV of 0.83, and accuracy of 0.89, all exceeding those of individual predictors (**Figure 4d**).

For differentiating adenoma from hyperplasia, although the AUC of the combined detection of ([Ca]×[Cl])/P and ALP (0.677, 95% CI = 0.563–0.790) remained higher than that of the single indicators, the overall diagnostic performance was relatively low (**Figure 4e**). When the cut-off values for ([Ca]×[Cl])/P and ALP were 351.65 mmol/L and 118.5 U/L, respectively, the PPV, NPV, and accuracy were 0.84, 0.50, and 0.69 (**Figure 4f**).

## 4 Discussion

This study demonstrated a significantly higher number of female patients with PHPT compared to males, with a median age of onset consistent with the findings of Kamal Govind et al. (10). Both the HPHPT and NPHPT groups were predominantly female, aligning with the results reported by T. Jiang et al. (11).The male-to-female ratio of PHPT in China, North America, and Western Europe is 1:3 (12), particularly common among postmenopausal women. The core mechanism is mainly related to decreased estrogen levels after menopause and an aggravated imbalance in bone metabolism, which exacerbates parathyroid dysfunction (3).

In this cohort, the overall proportion of adenomas among patients with PHPT was 73.33%, while hyperplasia accounted for 26.27%. The adenoma rate was similar to the 75%–85% range reported by Sun et al. (13); however, the hyperplasia rate observed in this study was relatively higher. This increase may be attributed to enhanced clinical awareness and the widespread use of multi-channel automatic analyzers and neck ultrasound, which improves the detection of incidental parathyroid lesions (6, 14). The overall proportion of single-gland lesions in this study was 83.70%, consistent with the findings of Tamski et al. (15) and the recommendations from the American Association of Clinical Endocrinologists (AACE) and the American Association of Endocrine Surgeons (AAES) (16). Moreover, adenomas and single-gland lesions predominated in both HPHPT and NPHPT groups compared to those of hyperplasia and multi-glandular lesions, reflecting similar pathological features in the two subtypes as reported by Yanling Yu et al. (4). Notably, glands in the NPHPT group were larger, potentially due to compensatory hyperplasia from prolonged mild PTH stimulation. However, clinicians should remain vigilant for malignancy risk in patients with PHPT and glandular masses exceeding 3.0 cm (17). In line with the WHO classification criteria for parathyroid tumors (5), vigilance should also be paid to the possibility of atypical parathyroid tumors in such large-volume gland lesions. As borderline tumors, they exhibit carcinoma-associated histological features despite the absence of definitive evidence of invasion or metastasis. Both atypical parathyroid tumors and parathyroid carcinomas are rare subtypes that require careful clinical differentiation.

The overall comparison of biochemical results indicated that patients with NPHPT exhibited better biochemical characteristics than those with HPHPT, consistent with the findings of Yankova et al. (18). This suggests that biochemical indicators can be utilized to evaluate disease severity. Additionally, the ALP level in the NPHPT group was higher than that in the control group, indicating that abnormal bone metabolism may already exist even when serum calcium levels are within the normal range. Notably, the preoperative ALP level in the HPHPT group was significantly higher than that in the NPHPT group, and the postoperative decreases in PTH and calcium levels in the HPHPT group (95.10% and 17.96%, respectively) were significantly greater than those in the NPHPT group (81.13% and 12.86%). Previous studies reported a negative correlation between preoperative ALP and postoperative serum calcium levels—higher preoperative ALP corresponds to lower postoperative serum calcium (19). Thus, preoperative ALP may serve as an important factor contributing to the differences in postoperative calcium metabolism recovery between the two groups and can be used as a key reference indicator for the clinical preoperative evaluation of the risk of postoperative hypocalcemia, providing a basis for tailored postoperative calcium supplementation regimens. Both primary and secondary hyperparathyroidism can present with elevated PTH, but differ significantly in pathophysiology. Secondary hyperparathyroidism is mostly a compensatory change caused by chronic renal injury, whereas primary hyperparathyroidism results from autonomous parathyroid dysfunction. The differences in ALP and ([Ca]×[Cl])/P in this study reflect distinct features in bone metabolism and calcium-phosphate homeostasis, providing clinical reference.

This study found that patients with PHPT exhibited more pronounced abnormalities in vitamin D metabolism, greater alterations in urinary calcium excretion, and lower bone mineral density compared with those with NPHPT. Moreover, serum calcium levels were closely associated with the severity of the above abnormalities. These results are consistent with the findings reported by Pelineagră OE et al. and Schaffler-Schaden D et al. (20, 21); Yankova et al. (18)also pointed out in their study that the differences in metabolic indicators of PHPT patients are mainly related to serum calcium level rather than pathological type. There were no significant differences in metabolic and bone indicators between patients with parathyroid adenoma and hyperplasia, suggesting that the pathological subtype is not the main factor determining metabolic and bone manifestations. It should be noted that, as a real-world observational study, some patients did not undergo certain specific examinations, which may have a certain impact on the accuracy of the AUC results. Further studies with larger populations and multi-center designs are warranted to validate our findings in the future. Therefore, routine detection of vitamin D, urinary calcium and bone mineral density in PHPT patients, and systematic bone metabolism evaluation can help in disease diagnosis, staging and individualized treatment, so as to improve the long-term prognosis of patients.

In this study, the AUC of the Ca/P ratio for diagnosing PHPT was 0.960, exceeding the AUC of 0.813 reported by Wright et al. (22). The cut-off value for differential diagnosis in this study was 2.57, which was similar to the cut-off value of 2.55 reported by Wright et al. (22) and Madeo et al. (23), indicating that the Ca/P ratio demonstrates good stability and practicality for diagnosing PHPT. Further analysis revealed that the AUC of the ([Ca]×[Cl])/P ratio for diagnosing PHPT reached 0.960, with the efficacy further improved to 0.983 when combined with ALP. The combined detection also showed strong performance in differentiating HPHPT from NPHPT, outperforming single indicators, such as Ca/P, Cl/P, ALP, and even PTH. The possible explanation is that PTH reduces serum phosphorus by inhibiting phosphorus reabsorption in the renal proximal tubules, while simultaneously promoting calcium reabsorption in the intestines and kidneys, thereby increasing serum calcium levels. Additionally, PTH enhances chloride reabsorption in the renal proximal tubules, leading to increased serum chloride (24). Nevertheless, although previous studies have demonstrated the auxiliary diagnostic value of the ([Ca]×[Cl])/P ratio in identifying PHPT (4, 25), the inherent correlations among its constituent variables may lead to information overlap and thus overestimate the diagnostic performance. Notably, elevated ALP levels reflect enhanced bone turnover induced by PTH, which is positively correlated with calcium and phosphorus metabolism disorders and indirectly reflects bone damage associated with PHPT (26). Therefore, the combined diagnosis of ([Ca]×[Cl])/P ratio and ALP may serve as an important method for PHPT screening and for differentiating between HPHPT and NPHPT.

Interestingly, this study found that the combined indicator of ([Ca]×[Cl])/P ratio and ALP demonstrated higher diagnostic efficacy than PTH in differentiating adenomas from hyperplasia. Although adenomas and hyperplasia differ in pathological morphology, both are characterized by excessive PTH secretion. The intensity of PTH secretion and its effects on metabolism and bone turnover overlap considerably, resulting in the limited diagnostic efficacy of PTH. Although the overall diagnostic performance of the combined indicator remained relatively weak, it may capture subtle differences in metabolic and bone turnover profiles that are not reflected by PTH alone, thus providing auxiliary value for clinical evaluation.

This study has certain limitations. First, this single-center retrospective study with a small sample may be subject to selection bias, and multicenter validation is required given the lack of external verification for the cutoff values. Second, all patients in this study were surgically treated and histopathologically confirmed, which may introduce spectrum bias such that this subgroup may not represent the broader PHPT population and may overestimate the diagnostic performance derived from ROC analysis. Third, due to the retrospective design of the study, only some participants had available key data including vitamin D status, 24-hour urinary calcium, and bone mineral density (BMD), which greatly limits the interpretation and generalizability of the biochemical indicators and may affect the reliability and comprehensiveness of the study conclusions. Finally, long-term postoperative follow-up was not performed; therefore, the long-term diagnostic stability and prognostic value of the combined indicator could not be verified, making it difficult to support extended management applications such as postoperative monitoring and early disease warning.

## 5 Conclusions

In conclusion, combined detection of ([Ca]×[Cl])/P and ALP exhibits favorable efficacy in diagnosing PHPT and differentiating HPHPT from NPHPT with high sensitivity and specificity. This method is slightly affected by fluctuating and borderline serum calcium levels, and overcomes the limitation of insufficient popularization of PTH detection at grassroots level. With simple operation and low cost, it is expected to serve as an effective tool for early screening in primary medical institutions. Although its diagnostic performance in distinguishing adenomas from hyperplasia is limited, it provides valuable supplementary information. Overall, this approach may provide a useful reference for preoperative decision-making in PHPT and deserves further validation for future clinical application and translation.

## Data Availability

The original contributions presented in the study are included in the article/**Supplementary Material**. Further inquiries can be directed to the corresponding author.

## References

[1] Alnajmi RAY Ali DS Khan AA . Persistence and recurrence of primary hyperparathyroidism. Best Pract Res Clin Endocrinol Metab. (2025) 39:101986. doi: 10.1016/j.beem.2025.101986 40074600

[2] El-Hajj Fuleihan G Chakhtoura M Cipriani C Eastell R Karonova T Liu JM . Classical and nonclassical manifestations of primary hyperparathyroidism. J Bone Mineral Research: Off J Am Soc For Bone Mineral Res. (2022) 37:2330–50. doi: 10.1002/jbmr.4679 36245249

[3] Chinese Medical Association Society of Osteoporosis and Bone Mineral Research, Metabolic Bone Disease Group of Chinese Society of Endocrinology . Guidelines for the diagnosis and treatment of primary hyperparathyroidism. Chin J Osteoporosis Bone Mineral Res. (2014) 7:187–98. doi: 10.1016/s0140-6736(80)91791-2

[4] Yu Y Qiu J Chuan F Feng Z Long J Zhou B . The Ca∗Cl/P ratio: a novel and more appropriate screening tool for normocalcaemic or overt primary hyperparathyroidism. Endocrine Practice: Off J Am Coll Endocrinol Am Assoc Clin Endocrinologists. (2024) 30:231–8. doi: 10.1016/j.eprac.2023.12.004 38086525

[5] Erickson LA Mete O Juhlin CC Perren A Gill AJ . Overview of the 2022 WHO classification of parathyroid tumors. Endocr Pathol. (2022) 33:64–89. doi: 10.1007/s12022-022-09709-1 35175514

[6] Bilezikian JP Khan AA Silverberg SJ Fuleihan GE Marcocci C Minisola S . Evaluation and management of primary hyperparathyroidism: summary statement and guidelines from the fifth international workshop. J Bone Mineral Research: Off J Am Soc For Bone Mineral Res. (2022) 37:2293–314. doi: 10.1002/jbmr.4677 36245251

[7] Zhu CY Sturgeon C Yeh MW . Diagnosis and management of primary hyperparathyroidism. JAMA. (2020) 323:1186–7. doi: 10.1001/jama.2020.0538 32031566

[8] He QQ Tian W . Chinese expert consensus on surgical practice of secondary hyperparathyroidism in chronic kidney disease (2021 edition). Chin J Pract Surg. (2021) 41:841–8. doi: 10.19538/j.cjps.issn1005-2208.2021.08.01

[9] Marcocci C Cetani F . Clinical practice. Primary hyperparathyroidism. N Engl J Med. (2011) 365:2389–97. doi: 10.1007/978-3-030-84367-0_21 22187986

[10] Govind K Paruk IM Motala AA . Characteristics, management and outcomes of primary hyperparathyroidism from 2009 to 2021: a single centre report from South Africa. BMC Endocr Disord. (2024) 24:53. doi: 10.1186/s12902-024-01583-8 38664758 PMC11044279

[11] Jiang T Yao XA Wei BB Chang H . The influence of gender on clinical manifestations of primary hyperparathyroidism. Zhonghua Nei Ke Za Zhi. (2018) 57:753–5. doi: 10.3760/cma.j.issn.0578-1426.2018.10.01 30293337

[12] Yanevskaya LG Karonova T Sleptsov IV Boriskova ME Bakhtiyarova AR Chernikov RA . Clinical phenotypes of primary hyperparathyroidism in hospitalized patients who underwent parathyroidectomy. Endocrine Connections. (2021) 10:248–55. doi: 10.1530/ec-20-0515 33416513 PMC7983481

[13] Sun B Guo B Wu B Kang J Deng X Zhang Z . Characteristics, management, and outcome of primary hyperparathyroidism at a single clinical center from 2005 to 2016. Osteoporosis International: A J Established as Result Cooperation Between Eur Foundation For Osteoporosis Natl Osteoporosis Foundation USA. (2018) 29:635–42. doi: 10.1007/s00198-017-4322-7 29198075

[14] Minisola S Arnold A Belaya Z Brandi ML Clarke BL Hannan FM . Epidemiology, pathophysiology, and genetics of primary hyperparathyroidism. J Bone Mineral Research: Off J Am Soc For Bone Mineral Res. (2022) 37:2315–29. doi: 10.1002/jbmr.4665 36245271 PMC10092691

[15] Tamski J Hakala T Huhtala H Metso S . Clinical characteristics and outcomes of patients operated for primary hyperparathyroidism at Tampere University Hospital in 2017-2018. Scandinavian J Surgery: SJS: Off Organ For Finnish Surg Soc Scandinavian Surg Soc. (2024) 113:254–60. doi: 10.1177/14574969241228409 38433618

[16] AACE/AAES Task Force on Primary Hyperparathyroidism . The American Association of Clinical Endocrinologists and the American Association of Endocrine Surgeons position statement on the diagnosis and management of primary hyperparathyroidism. Endocrine Practice: Off J Am Coll Endocrinol Am Assoc Clin Endocrinologists. (2005) 11:49–54. doi: 10.4158/ep.11.1.49 16033736

[17] Bae JH Choi HJ Lee Y Moon MK Park YJ Shin CS . Preoperative predictive factors for parathyroid carcinoma in patients with primary hyperparathyroidism. J Korean Med Sci. (2012) 27:890–5. doi: 10.3346/jkms.2012.27.8.890 22876055 PMC3410236

[18] Yankova I Lilova L Petrova D Dimitrova I Stoynova M Shinkov A . Biochemical characteristics and clinical manifestation of normocalcemic primary hyperparathyroidism. Endocrine. (2024) 85:341–6. doi: 10.1007/s12020-024-03768-6 38489132

[19] Mu Y Zhao Y Zhao J Zhao Q Zhang Y Li Y . Factors influencing serum calcium levels and the incidence of hypocalcemia after parathyroidectomy in primary hyperparathyroidism patients. Front Endocrinol. (2023) 14:1276992. doi: 10.3389/fendo.2023.1276992 38116316 PMC10728860

[20] Pelineagră OE Golu I Bala M Amzăr D Plotuna I Popa O . Unraveling the paradox of vitamin D status in primary hyperparathyroidism: an incidental finding or an unexpected consequence? Int J Mol Sci. (2025) 26(9):4434. doi: 10.3390/ijms26094434 40362670 PMC12072446

[21] Schaffler-SChaden D Schweighofer-Zwink G Hehenwarter L van der Zee-Neuen A Flamm M Beheshti M . Bone mineral density and first line imaging with [(18)F]fluorocholine PET/CT in normocalcemic and hypercalcemic primary hyperparathyroidism: results from a single center. Diagnostics (Basel Switzerland). (2024) 14(22):2466. doi: 10.3390/diagnostics14222466 39594132 PMC11592530

[22] Wright C King D Small M Gibson C Gardner R Stack BC . The utility of the Cl:PO4 ratio in patients with variant versions of primary hyperparathyroidism. Otolaryngology--Head Neck Surgery: Off J Am Acad Otolaryngology-Head Neck Surg. (2021) 164:308–14. doi: 10.1177/0194599820947009 32746759

[23] Madeo B De Vincentis S Repaci A Altieri P Vicennati V Kara E . The calcium-to-phosphorous (Ca/P) ratio in the diagnosis of primary hyperparathyroidism and hypoparathyroidism: a multicentric study. Endocrine. (2020) 68:679–87. doi: 10.1007/s12020-020-02276-7 32236819

[24] Yin M Liu Q Wang Q He Y Song H Nie X . Diagnostic performance of the calcium/phosphate ratio for primary hyperparathyroidism in southwest China. Endocrine Connections. (2021) 10:1387–92. doi: 10.1530/ec-21-0267 34559066 PMC8558886

[25] Şah Ünal FT Demir Ö Emral R Gökçay Canpolat A . Biochemical ratios for predicting primary hyperparathyroidism: revisiting simple yet powerful diagnostic tools. Clin Endocrinol. (2026) 104:19–26. doi: 10.1111/cen.70052 41133987

[26] Wang Q Li X Chen H Yu H Li L Yin J . The chloride/phosphate ratio combined with alkaline phosphatase as a valuable predictive marker for primary hyperparathyroidism in Chinese individuals. Sci Rep. (2017) 7:4868. doi: 10.1038/s41598-017-05183-6 28687737 PMC5501820

