# Supplementary material for: Diagnostic efficacy of ([Ca]×[Cl])/P combined with ALP in the diagnosis and subtype differentiation of primary hyperparathyroidism
Source: Front Endocrinol (Lausanne). 2026 Jun 5;17:1790881. doi: 10.3389/fendo.2026.1790881 (PMC13278938; doi:10.3389/fendo.2026.1790881)
Supplement: Supplementary file 1 [file Table1.docx]

Supplement Table 1 Baseline characteristics and laboratory parameters in patients with primary hyperparathyroidism and secondary hyperparathyroidism

|  | HPHPT(n=77) | NPHPT(n=58) | Secondary hyperparathyroidism(n=135) | P |
| --- | --- | --- | --- | --- |
| **Gender(Male/Female)** |  |  |  | 0.467 |
| Male n(%) | 24(31.17) | 13(22.41) | 41(30.37) |  |
| Female n(%) | 53(68.83) | 45(77.59) | 94(69.63) |  |
| Median age (mean,sd), year | 53.45±12.17 | 53.43±11.34 | 54.52±8.94 | 0.834 |
| BMI (IQRa), kg/m2 | 23.09±6.06 | 24.58±3.96 | 23.50±3.61 | 0.141 |
| Hypertension n(%) | 26(33.76) | 18(31.03) | 114(84.44)^#+^ | **<0.001** |
| Diabetes **n(%)** | 9(11.69) | 7(12.07) | 27(77.14) | 0.187 |
| Kidney stones **n(%)** | 15(19.48) | 5(8.62) | 7(5.19) | **0.004** |
| **Maximum diameter (mm)** | 21.68±12.72 | 21.44±7.46 | / | 0.282 |
| **Lesions** |  |  |  | 0.494 |
| Single-gland lesion | 63(81.82) | 50(86.21) | / |  |
| Multiglandular lesions | 14(18.18) | 8(13.79) | / |  |
| **Pathological results** |  |  |  | **0.003** |
| Adenoma n(%) | 64(83.12) | 35(60.34) | / |  |
| Hyperplasia n(%) | 13(16.88) | 23(39.66) | / |  |
| **Laboratory indicators** |  |  |  |  |
| PTH (pg/mL) | 263.3(189.0-531.0) | 129.3 (92.8-186.6)* | 1145.0(309.0-1991.0)^#+^ | **<0.001** |
| WBC (×10^9/L) | 6.1(4.8-7.7) | 6.0(4.8-7.0) | 5.96(5.0-7.3) | 0.700 |
| RBC (×10^12/L) | 4.54(4.15-4.89) | 4.57 (4.16-4.90) | 3.77(3.28-4.10)^#+^ | **<0.001** |
| PLT (×10^9/L) | 231.0(195.0-298.0) | 238.5 (208.8-298.8) | 216.0(167.0-279.0)^#+^ | **<0.001** |
| ALT (U/L) | 18.0(14.1-30.0) | 19.5 (13.8-34.5) | 11.0(8.0-16.0)^#+^ | **<0.001** |
| AST (U/L) | 22.0(17.0-26.5) | 23.0 (19.0-28.0)* | 15.0(12.0-20.0)^#+^ | **<0.001** |
| ALP (U/L) | 141.0(107.0-234.5) | 102.0(81.8-119.0)* | 184.0(115.0-351.0)^+^ | **<0.001** |
| TP (g/L) | 72.2(68.7-75.2) | 75.2 (70.4-77.7) | 68.1(62.8-74.1)^#+^ | **<0.001** |
| ALB (g/L) | 44.1(41.8-45.7) | 45.8 (43.6-48.0)* | 38.2(34.7-41.8)^#+^ | **<0.001** |
| eGFR(mL/min/1.73m²) | 98.0（77.0-110.5） | 100.0 (91.0-110.8) | 5.0（4.0-6.0）#+ | **<0.001** |
| K (mmol/L) | 4.16(3.94-4.44) | 4.15 (4.00-4.38) | 4.46(3.97-4.88)^#+^ | **<0.001** |
| NA (mmol/L) | 140.2(138.7-141.6) | 140.2 (138.4-141.7) | 139.0(137.2-141.3) | **<0.001** |
| CL (mmol/L) | 107.6(105.6-109.5) | 105.7(104.8-107.9)* | 99.6(17.5-102.8)^#+^ | **<0.001** |
| P(mmol/L) | 0.73(0.64-0.82) | 0.90 (0.76-1.02)* | 1.76(1.48-2.12)^#+^ | **<0.001** |
| Albumin-corrected calcium(mmol/L) | 2.84(2.75-3.02) | 2.49(2.37-2.59)* | 2.37(2.23-2.53)^#+^ | **<0.001** |
| Ca/P | 3.90(3.42-4.74) | 2.82(2.38-3.29)* | 1.36(1.14-1.60)^#+^ | **<0.001** |
| CL/P | 145.38(129.75-169.24) | 120.63 (101.92-139.37)* | 57.17(46.23-67.17)^#+^ | **<0.001** |
| ([Ca]×[Cl])/P(mmol/L) | 428.13(367.13-509.84) | 302.25(250.54-350.70)* | 136.0(113.10-157.07)^#+^ | **<0.001** |
| Postoperative 0-1 day PTH (pg/mL) | 12.9(7.8-25.1) | 24.4 (13.2-36.2) | / | **<0.001** |
| Postoperative 0-1 day calcium (mmol/L) | 2.33(2.20-2.46) | 2.17(2.11-2.29) | / | **<0.001** |

**Note:** Data are expressed as median (IQR), n (%), or mean ± sd. *: P < 0.05 (HPHPT vs. NPHPT); #: P<0.05 (HPHPT vs. Secondary hyperparathyroidism group); +: P<0.05 (NPHPT vs. Secondary hyperparathyroidism group); P: P value for comparison among HPHPT, NPHPT, and Secondary hyperparathyroidism groups; BMI: Body Mass Index; PTH: Parathyroid hormone; WBC: White Blood Cell; RBC: Red Blood Cell; PLT: Platelet; ALT: Alanine Aminotransferase; AST: Aspartate Aminotransferase; ALP: Alkaline Phosphatase; GGT: Gamma-Glutamyl Transferase; TP: Total Protein; ALB: Albumin; eGFR: Estimated glomerular filtration rate was calculated using the CKD-EPI equation based on serum creatinine. Ca/P: albumin-Corrected Calcium to Phosphorus Ratio; Cl/P: chloride to Phosphorus Ratio; ([Ca]×[Cl])/P: the ratio of the product of albumin-corrected calcium and chloride to phosphorus.
